# Supplementary material for: Development of a broad-spectrum epitope-based vaccine against Streptococcus pneumoniae
Source: PLoS One. 2025 Jan 16;20(1):e0317216. doi: 10.1371/journal.pone.0317216 (PMC11737669; doi:10.1371/journal.pone.0317216)
Supplement: S1 Table — (DOCX) [file pone.0317216.s001.docx]

**Table S1.**: Antigenicity prediction for 23 immunogenic proteins of *S. pneumoniae* by VaxiJen server. The Bacterial subcellular location of the protein assessed by PsortB.

| **Serial No** | **Proteins name** | **Accession number** | **Vaxijen score** | **PsortB (score)** |
| --- | --- | --- | --- | --- |
| 01. | Choline-binding protein A (cbpA) | CJD51254.1 | 0.7721 | Extracellular (9.73) |
| 02. | Pneumococcal surface protein A (PspA) | VMA87220.1 | 0.6565 | Extracellular (9.73) |
| 03. | Pneumococcal histidine triad protein B (phtB) | QEY10698.1 | 0.6099 | Cytoplasmic Membrane (4.60) |
| 04. | LPXTG-anchored neuraminidase NanA | TVW37091.1 | 0.6072 | Cell wall (10.00) |
| 05. | Pneumococcal histidine triad protein D (phtD) | AVN86012.1 | 0.6017 | Cell wall (2.48) |
| 06. | Pneumococcal histidine triad protein (phtE) | WP_170081610.1 | 0.5938 | Extracellular (3.33) |
| 07. | Choline-binding protein D (cbpD) | BBG82645.1 | 0.5893 | Extracellular (3.33) |
| 08. | Pneumococcal lipoprotein (piaA) | VSP17585.1 | 0.5804 | Cell wall (3.33) |
| 09. | immunoglobulin A1 protease (IgA1) | WP_219938683.1 | 0.5770 | Cell wall (10.00) |
| 10. | Autolysin (LytA) | VDG79395.1 | 0.5750 | Extracellular (9.60) |
| 11. | Pullulanase (spuA) | AAG33958.1 | 0.5616 | Cell wall (9.99) |
| 12. | Neuraminidase B (nanB) | WP_288176786.1 | 0.5486 | Extracellular (8.19) |
| 13. | Streptokinase (stkP) | SBO71241.1 | 0.5478 | Cytoplasmic Membrane (9.96) |
| 14. | Zinc metalloproteinase C (zmpC) | WP_224347781.1 | 0.5401 | Cell wall (9.94) |
| 15. | Autolysin C (lytC) | WP_170081610.1 | 0.5084 | Extracellular (9.13) |
| 16. | Zinc metalloproteinase B (zmpB) | VSS97198.1 | 0.5066 | Cell wall (9.94) |
| 17. | pneumococcal surface adhesin A (PsaA) | AF459738_1 | 0.5027 | Cytoplasmic Membrane (9.99) |
| 18. | Pneumolysin (Ply) | VTY30387.1 | 0.4734 | Extracellular (9.60) |
| 19. | Neuraminidase A (nanA) | ARD36335.1 | 0.4336 | Cytoplasmic Membrane (9.55) |
| 20. | Hyaluronidase | AAA53685.1 | 0.3994 | Cell wall (9.49) |
| 21. | Autolysin B (lytB) | AVN85978.1 | 0.3798 | Extracellular (9.73) |
| 22. | Phosphorylcholine (ChoP) | CGF52948.1 | 0.3256 | Cytoplasmic (7.50) |
| 23. | fibronectin-binding protein (PavA) | WP_288177290.1 | 0.1992 | Cytoplasmic (7.50) |
