# Supplementary material for: Development of a broad-spectrum epitope-based vaccine against Streptococcus pneumoniae
Source: PLoS One. 2025 Jan 16;20(1):e0317216. doi: 10.1371/journal.pone.0317216 (PMC11737669; doi:10.1371/journal.pone.0317216)
Supplement: S2 Table — (DOCX) [file pone.0317216.s002.docx]

**Table 1. The predicted B cell epitopes of CbpA.**

| **ABCpred (CbpA)** | | | | | | | | | |
| --- | --- | --- | --- | --- | --- | --- | --- | --- | --- |
| Sequence | **Score** | **Sequence** | | **Score** | | **Sequence** | **Score** | **Sequence** | **Score** |
| RGALGEQATPDKKEND | 0.93 | | NGSWYYLNANGDMATG | | 0.87 | PPKTEKPAQPSTPKTG | 0.80 | HYSIRKFSIGVASVVV | 0.74 |
|  |  | |  | |  |  |  |  |  |
| LSRIQTEYLYKLKVNG | 0.92 | | NGSWYYLNANGDMATG | | 0.87 | RKSRGKRGALGEQATP | 0.80 | VGEETLPSPSLKLGKK | 0.73 |
|  |  | |  | |  |  |  |  |  |
| NGSWYYLNANGAMATG | 0.92 | | NGSWYYLNANGDMATG | | 0.87 | ANGDMATGWVKDGDTW | 0.79 | SRNEGTINQAKAKVES | 0.73 |
|  |  | |  | |  |  |  |  |  |
| NGSWYYLNANGAMATG | 0.92 | | PKPEKPAEQPKAEKTD | | 0.87 | GSMATGWLQNNGSWYY | 0.79 | VAEAEKKVEEAKKKAE | 0.73 |
|  |  | |  | |  |  |  |  |  |
| NGMWYFYNTDGSMATG | 0.92 | | MATGWAKVNGSWYYLN | | 0.86 | GSMATGWLQNNGSWYY | 0.79 | VAEAEKKVEEAKKKAK | 0.72 |
|  |  | |  | |  |  |  |  |  |
| KAEEEAKRRADAKEQD | 0.92 | | MATGWAKVNGSWYYLN | | 0.86 | GVRSENTPTVTSSGQD | 0.79 | TKKELDAVFEQFKKDT | 0.72 |
|  |  | |  | |  |  |  |  |  |
| NGSWYYLNSNGAMATG | 0.91 | | SRLSEIKLDRRKHTQN | | 0.86 | RLTQQQPPKTEKPAQP | 0.78 | ADEVKSHIESRLSEIK | 0.70 |
|  |  | |  | |  |  |  |  |  |
| KKKAEDQKEEDRRNYP | 0.90 | | PAPQPEKPAPKPEKPA | | 0.85 | TNTYKTLELEIAESDV | 0.78 | AEQPKAEKTDDQQAEE | 0.65 |
|  |  | |  | |  |  |  |  |  |
| KEEAKEPRDEEKIKQA | 0.89 | | ELVKEEANESRNEGTI | | 0.85 | RADAKEQDESKKRKSR | 0.78 | ASKSERKVHYSIRKFS | 0.65 |
|  |  | |  | |  |  |  |  |  |
| KKKAKDQKEEDRRNYP | 0.89 | | SGAMKASQWFKVSDKW | | 0.84 | SKKAEATRLENIKTDR | 0.77 | YKLKVNGLEEKSKAEL | 0.65 |
|  |  | |  | |  |  |  |  |  |
| GDTWYYLEASGAMKAS | 0.88 | | SGAMKASQWFKVSDKW | | 0.84 | QKEEDRRNYPTNTYKT | 0.77 | ELEIAESDVKVKEAEL | 0.64 |
|  |  | |  | |  |  |  |  |  |
| NGSWYYLNANGSMATG | 0.88 | | ATGWLQYNGSWYYLNS | | 0.84 | TITYKTLDLEIAEFDV | 0.77 | DLEIAEFDVKVKEAEL | 0.62 |
|  |  | |  | |  |  |  |  |  |
| GDTWYYLEASGAMKAS | 0.88 | | RRSEEEYNRLTQQQPP | | 0.83 | DTLSTEPEKKVAEAEK | 0.77 | DISKKYADEVKSHIES | 0.61 |
|  |  | |  | |  |  |  |  |  |
| NGSWYYLNANGSMATG | 0.88 | | TGWVKDGDTWYYLEAS | | 0.82 | DKKENDAKSSDSSVGE | 0.76 | KVESEKAEATRLKKIK | 0.60 |
|  |  | |  | |  |  |  |  |  |
| LGGVVHAEGVRSENTP | 0.88 | | SDKWYYVNSNGAMATG | | 0.82 | ANGAMATGWLQNNGSW | 0.75 | PRDEEKIKQAKAKVES | 0.58 |
|  |  | |  | |  |  |  |  |  |
| GALAVNTTVDGYGVNA | 0.87 | | TGWVKDGDTWYYLEAS | | 0.82 | KAAEEDKVKEKPAEQP | 0.75 | IKQAKAKVESKKAEAT | 0.57 |
|  |  | |  | |  |  |  |  |  |
| ANGSMATGWVKDGDTW | 0.87 | | SDKWYYVNGSGALAVN | | 0.81 | DRRNYPTITYKTLDLE | 0.75 | PSPSLKLGKKVAEAEK | 0.55 |
|  |  | |  | |  |  |  |  |  |
| NGSWYYLNANGDMATG | 0.87 | | TGWKQENGMWYFYNTD | | 0.81 | KSKAELTSKTKKELDA | 0.75 | LDRRKHTQNFNLNIKL | 0.53 |
|  |  | |  | |  |  |  |  |  |
| NGSWYYLNANGDMATG | 0.87 | | TVTSSGQDISKKYADE | | 0.81 | QQAEEDYARRSEEEYN | 0.74 |  |  |
|  |  | |  | |  |  |  |  |  |
| NGSWYYLNANGDMATG | 0.87 | | KAEEEAKRKAAEEDKV | | 0.81 | EKPAEQPQPAPAPQPE | 0.74 |  |  |

| **LBTope (CbpA)** | | |
| --- | --- | --- |
| Sequence | Sequence | Sequence |
| [KKYADEVKSHIESRLSEIK](https://webs.iiitd.edu.in/raghava/lbtope/vrindex/lbtope.php?ran=57456) | [KKVEEAKKKAEDQK](https://webs.iiitd.edu.in/raghava/lbtope/vrindex/lbtope.php?ran=57456) | [LGKKVAEAEKKV](https://webs.iiitd.edu.in/raghava/lbtope/vrindex/lbtope.php?ran=57456) |
| [KVNGL](https://webs.iiitd.edu.in/raghava/lbtope/vrindex/lbtope.php?ran=57456) | [GALGEQATPDKKENDAKS](https://webs.iiitd.edu.in/raghava/lbtope/vrindex/lbtope.php?ran=57456) | [KKKAKDQKEEDRRNYPTN](https://webs.iiitd.edu.in/raghava/lbtope/vrindex/lbtope.php?ran=57456) |
| [SKTKKELDAVF](https://webs.iiitd.edu.in/raghava/lbtope/vrindex/lbtope.php?ran=57456) | [PSPSLK](https://webs.iiitd.edu.in/raghava/lbtope/vrindex/lbtope.php?ran=57456) | [GYGVNAN](https://webs.iiitd.edu.in/raghava/lbtope/vrindex/lbtope.php?ran=57456) |

| **Emini surface accessibility Prediction (CbpA)** | | |
| --- | --- | --- |
| Sequence | **Sequence** | **Sequence** |
| KSERKV | IKTDREKAEEEAKRRADAKEQDESKKRKSRG | DKVKEKPAEQPQ |
| DRRKHT | ATPDKKENDAKSS | PQPEKPAPKPEKPAEQPKAEKTDDQQAE |
| SKTKKE | AEKKVEEAKKKAKDQKEEDRRNYPTNTYK | EKDYDAAVKKSEAAKKDYETAKKKAEDAQKK |
| AEKKVEEAKKKAEDQKEEDRRN | EEAKEPRDEEKIK | TGWKQE |
| EANESRN | IKTDRKKAEEEAKRKAAE |  |

**Ellipro (CbpA)**

| Sequence | Score | Sequence | Score |
| --- | --- | --- | --- |
| VEEAKKKAKDQKEEDRRNYPTNTYKTLELEIAESDVKVKEAELELVKEEAKEPRDEEKIKQAKAKVESKKAEATRLENIKTDRKKAEEEAK | 0.818 | NYPTITYKTLDLEIAEFDVKVKEAELELVKEEANESRNEGTINQAKAKVESEKAEATRLKKIKTDREKA | 0.723 |
| DMATGWAKVNGSWYYLNANGDMATGWAKVNGSWYYLNANGSMATGWVKDGDTWYYLEASGAMKASQWFKVSDKWYYVNGSGALAVNTTVDGYGVNANGEWVN | 0.766 | TGWLQYNGSWYYLN | 0.563 |
| EKPAPKPEKPAEQPKAEKTDDQQAEEDYARRSEEEYNRLTQQQPPKTEKPAQPSTPKTGWKQENGMWYFYNTDGSMATGWLQNNGSWYYLNANGAMATGWLQNNGSWYYLNANGSMAT | 0.732 | LNANGAMATG | 0.544 |

**Table 2. The predicted B cell epitopes of PspA.**

| **ABCpred (PspA)** | | | | | | | |
| --- | --- | --- | --- | --- | --- | --- | --- |
| Sequence | **Score** | **Sequence** | **Score** | **Sequence** | **Score** | **Sequence** | **Score** |
| NGSWYYLNANGAMATG | 0.92 | KEVQQAYLAYLQASNE | 0.87 | AEKERKASEKIAEATK | 0.80 | EKDYDAAVKKSEAAKK | 0.73 |
|  |  |  |  |  |  |  |  |
| NGSWYYLNANGAMATG | 0.92 | DPEGKTQDELDKETAE | 0.86 | KSEAAKKDYETAKKKA | 0.80 | KVAELEEELSKLEDNL | 0.73 |
|  |  |  |  |  |  |  |  |
| NGMWYFYNTDGSMATG | 0.92 | DEEETPAPAPKPEKPA | 0.85 | VEDYIKEGLEEAIATK | 0.80 | AETKKKAEEATKEAEV | 0.73 |
|  |  |  |  |  |  |  |  |
| NGSWYYLNSNGAMATG | 0.91 | AEAKIKATTKKAELEK | 0.85 | EAAEKDLATKKAELAE | 0.80 | EAEAAFATIRTTIVVP | 0.73 |
|  |  |  |  |  |  |  |  |
| AEDAQKKYDEDQKKTE | 0.90 | SGAMKASQWFKVSDKW | 0.84 | KKSEEAAKEVEVEKNK | 0.80 | EQDAENEKKIDVLQNK | 0.70 |
|  |  |  |  |  |  |  |  |
| GFVASSPTFVRAEEAP | 0.90 | SGAMKASQWFKVSDKW | 0.84 | GSMATGWLQNNGSWYY | 0.79 | AYLQASNESQRKEADK | 0.70 |
|  |  |  |  |  |  |  |  |
| GSLAVNTTVDGYTVNE | 0.89 | EVEKNKILEQDAENEK | 0.84 | PAPKPEQPAPAPKTGW | 0.79 | YDEDQKKTEAKAEKER | 0.69 |
|  |  |  |  |  |  |  |  |
| GDTWYYLEASGAMKAS | 0.88 | RRSEEEYNRLTQQQPP | 0.83 | PVANQSKAEKDYDAAV | 0.79 | TPAPKPEKSADQQAEE | 0.67 |
|  |  |  |  |  |  |  |  |
| NGSWYYLNANGSMATG | 0.88 | PKPEQPTPAPKPEKSA | 0.83 | DGEYAEFYLEAAEKDL | 0.79 | ENLLSTLDPEGKTQDE | 0.67 |
|  |  |  |  |  |  |  |  |
| GDTWYYLEASGAMKAS | 0.88 | EKTQKALDTALNELGP | 0.83 | FVRAEEAPVANQSKAE | 0.77 | KAELEKAEAELENLLS | 0.67 |
|  |  |  |  |  |  |  |  |
| NGSWYYLNANGSMATG | 0.88 | TGWVKDGDTWYYLEAS | 0.82 | LEKEVAKLEKDVEGFK | 0.77 | DLEKGIADAEKTVADL | 0.67 |
|  |  |  |  |  |  |  |  |
| KKMILTSLASVAILGA | 0.88 | SDKWYYVNSNGAMATG | 0.82 | EGLEEAIATKQAELEK | 0.75 | EKTVADLEKEVAKLEK | 0.66 |
|  |  |  |  |  |  |  |  |
| ANGSMATGWVKDGDTW | 0.87 | TGWVKDGDTWYYLEAS | 0.82 | AEEATKEAEVAKKKSE | 0.75 | TIVVPEPSELAETKKK | 0.66 |
|  |  |  |  |  |  |  |  |
| ANGAMATGWAKVNGSW | 0.87 | PPKAEKPAPAPAPKPE | 0.82 | ASVAILGAGFVASSPT | 0.75 | ESQRKEADKKIKEATQ | 0.64 |
|  |  |  |  |  |  |  |  |
| ANGSMATGWVKDGDTW | 0.87 | SDKWYYVNGSGSLAVN | 0.81 | SEKIAEATKEVQQAYL | 0.74 | LSKLEDNLKVAETNNV | 0.60 |
|  |  |  |  |  |  |  |  |
| ANGAMATGWAKVNGSW | 0.87 | TGWKQENGMWYFYNTD | 0.81 | QQAEEDYARRSEEEYN | 0.74 | ELDKETAEAELNKKVE | 0.54 |
|  |  |  |  |  |  |  |  |
| TALNELGPDGDEEETP | 0.87 | PAPKPEKPAPAPAPKP | 0.81 | DKKIKEATQRKDEAEA | 0.74 |  |  |

| **LBTope (PspA)** | | |
| --- | --- | --- |
| Sequence | Sequence | Sequence |
| ADITKNIAGDKIDLH | YLEGQNEKGKEDPHA | PIGQDPHEYEPLPED |
| DITKNIAGDKIDLHS | LEGQNEKGKEDPHAW | IGQDPHEYEPLPEDV |
| VPIGQDPHEYEPLPE | EGQNEKGKEDPHAWL | GQDPHEYEPLPEDVK |

| **Emini surface accessibility Prediction (PspA)** | | |
| --- | --- | --- |
| Sequence | **Sequence** | **Sequence** |
| QSKAEKDY | AKKKSEEA | PAPKPEKPA |
| YDEDQKKTEAKA | DAENEK | PAPKPEQPTPAPKPEKSA |
| SNESQRKEADKKI | TTKKAE | QQAEEDYARRSEEEYNRLTQQQPPKAEKPA |
| ATQRKDE | EGKTQDELD | PAPKPEQP |
| ETKKKAEEATK | DGDEEET | TGWKQE |

**Ellipro (PspA)**

| Sequence | Score | Sequence | Score |
| --- | --- | --- | --- |
| SEAAKKDYETAKKKAEDAQKKYDEDQKKTEAKAEKERKA | 0.846 | SMATGWVKDGDTWYYLEASGAMKASQWFKVSDKWYYVNGSGSLAVNTTVDGYTVNENGEWV | 0.717 |
| KGIADAEKTVADLEKEVAKLEKDVEGFKESDGEYAEFYLEAAEKDLATKKAELAEAKIK | 0.791 | AELEEELSKLEDNLKVAETNNVEDYIKEGLE | 0.653 |
| PAPAPKPEQPAPAPKTGWKQENGMWYFYNTDGSMATGWLQNNGSWYYLNSNGAMATGWLQYNGSWYYLNANGAMAT | 0.724 |  |  |
