# Supplementary material for: Development of a broad-spectrum epitope-based vaccine against Streptococcus pneumoniae
Source: PLoS One. 2025 Jan 16;20(1):e0317216. doi: 10.1371/journal.pone.0317216 (PMC11737669; doi:10.1371/journal.pone.0317216)
Supplement: S3 Table — (DOCX) [file pone.0317216.s003.docx]

**Table 1.1:** Predicted CTL epitopes for CbpA after screening with NetCTL 1.2, TmHMM, Vaxijen, AlletrTop, ToxinPred server for Human alleles.

| **Epitope** | **TmHMM** | **Vaxijen** | **Allertop** | **ToxinPred** |
| --- | --- | --- | --- | --- |
| DRRKHTQNF | Outside | 0.9097 | Non-allergen | Non-toxin |
| IQTEYLYKL | Outside | 0.8331 | Non-allergen | Non-toxin |
| KVSDKWYYV | Outside | 1.2339 | Non-allergen | Non-toxin |
| RKHTQNFNL | Outside | 1.4515 | Non- allergen | Non-toxin |
| RNYPTNTYK | Outside | 0.6313 | Non- allergen | Non-toxin |
| RRNYPTNTY | Outside | 0.7645 | Non- allergen | Non-toxin |
| SERKVHYSI | Outside | 1.0488 | Non- allergen | Non-toxin |
| SNGAMATGW | Outside | 0.5211 | Non- allergen | Non-toxin |
| SVVVASLFL | Outside | 1.0053 | Non- allergen | Non-toxin |
| VASVVVASL | Outside | 0.4747 | Non- allergen | Non-toxin |
| WFKVSDKWY | Outside | 0.6617 | Non- allergen | Non-toxin |
| YLNANGAMA | Outside | 0.5612 | Non- allergen | Non-toxin |
| YYLEASGAM | Outside | 0.5396 | Non- allergen | Non-toxin |
| YYLNANGAM | Outside | 0.7736 | Non- allergen | Non-toxin |
| YYLNANGDM | Outside | 0.8878 | Non- allergen | Non-toxin |
| YYLNANGSM | Outside | 0.8656 | Non- allergen | Non-toxin |
| YYVNGSGAL | Outside | 0.6450 | Non- allergen | Non-toxin |

**Table 1.2:** Predicted CTL epitopes for CbpA after-1st filtration NetMHC server for mice alleles.

| **Epitope** | **Score (NetMHC 4.1)** |
| --- | --- |
| IQTEYLYKL | 0.353 |
| YYLEASGAM | 0.059 |
| YYLNANGAM | 0.135 |
| YYLNANGDM | 0.427 |
| YYLNANGSM | 0.076 |
| YYVNGSGAL | 0.024 |
| SERKVHYSI | 0.042 |
| IQTEYLYKL | 0.427 |

**Supplementary Table 2.1:** Predicted CTL epitopes for PspA after screening with NetCTL 1.2, TmHMM, Vaxijen, AlletrTop, ToxinPred, NetMHC server for Human alleles.

| **Epitopes** | **Transmembrane Helix** | **Antigenicity** | **Allergenicity** | **Toxicity** |
| --- | --- | --- | --- | --- |
| EVQQAYLAY | Outside | 0.6216 | Non-allergen | Non-toxin |
| KPAPAPAPK | Outside | 0.8288 | Non-allergen | Non-toxin |
| KVSDKWYYV | Outside | 1.2339 | Non-allergen | Non-toxin |
| SNGAMATGW | Outside | 0.5211 | Non-allergen | Non-toxin |
| WFKVSDKWY | Outside | 0.6617 | Non-allergen | Non-toxin |
| YLNANGAMA | Outside | 0.5612 | Non-allergen | Non-toxin |
| YYLEASGAM | Outside | 0.5396 | Non-allergen | Non-toxin |
| YYLNANGAM | Outside | 0.7736 | Non-allergen | Non-toxin |
| YYLNANGSM | Outside | 0.8656 | Non-allergen | Non-toxin |
| YYVNGSGSL | Outside | 0.7766 | Non-allergen | Non-toxin |

**Supplementary Table 2.2:** Predicted CTL epitopes for PspA after-2nd filtration, NetMHC server for Mice alleles.

| **Epitopes** | **Score (NetMHC 4.1)** |
| --- | --- |
| YYLEASGAM | 0.059 |
| YYLNANGAM | 0.135 |
| YYLNANGSM | 0.076 |
| YYVNGSGSL | 0.011 |
