# Supplementary material for: Development of a broad-spectrum epitope-based vaccine against Streptococcus pneumoniae
Source: PLoS One. 2025 Jan 16;20(1):e0317216. doi: 10.1371/journal.pone.0317216 (PMC11737669; doi:10.1371/journal.pone.0317216)
Supplement: S4 Table — (DOCX) [file pone.0317216.s004.docx]

**Table 1.1:**  Predicted HTL epitopes (CbpA) were shortlisted after assessment through TMHMM, VaxiJen v2.0, AllerTOP v.2.0, and ToxinPred servers.

| **Epitopes** | **Transmembrane Helix** | **Antigenicity** | **Allergenicity** | **Toxicity** |
| --- | --- | --- | --- | --- |
| AEGVRSENTPTVTSS | Outside | 1.4384 | Non-Allergen | Non-toxin |
| AKVNGSWYYLNANGD | Outside | 0.9052 | Non-Allergen | Non-toxin |
| ANGDMATGWAKVNGS | Outside | 0.5125 | Non-Allergen | Non-toxin |
| ASQWFKVSDKWYYVN | Outside | 0.4571 | Non-Allergen | Non-toxin |
| DKWYYVNGSGALAVN | Outside | 0.5698 | Non-Allergen | Non-toxin |
| DKWYYVNSNGAMATG | Outside | 0.6579 | Non-Allergen | Non-toxin |
| GGVVHAEGVRSENTP | Outside | 1.2685 | Non-Allergen | Non-toxin |
| GSWYYLNANGAMATG | Outside | 0.6041 | Non-Allergen | Non-toxin |
| GSWYYLNANGDMATG | Outside | 0.7856 | Non-Allergen | Non-toxin |
| GSWYYLNSNGAMATG | Outside | 0.5133 | Non-Allergen | Non-toxin |
| GVRSENTPTVTSSGQ | Outside | 1.4493 | Non-Allergen | Non-toxin |
| KQENGMWYFYNTDGS | Outside | 0.5411 | Non-Allergen | Non-toxin |
| KWYYVNGSGALAVNT | Outside | 0.8465 | Non-Allergen | Non-toxin |
| KWYYVNSNGAMATGW | Outside | 0.7151 | Non-Allergen | Non-toxin |
| LGGVVHAEGVRSENT | Outside | 1.196 | Non-Allergen | Non-toxin |
| NANGDMATGWAKVNG | Outside | 0.5491 | Non-Allergen | Non-toxin |
| NANGDMATGWVKDGD | Outside | 0.6769 | Non-Allergen | Non-toxin |
| NANGSMATGWVKDGD | Outside | 0.6742 | Non-Allergen | Non-toxin |
| PKTGWKQENGMWYFY | Outside | 0.4212 | Non-Allergen | Non-toxin |
| QWFKVSDKWYYVNGS | Outside | 0.5754 | Non-Allergen | Non-toxin |
| QWFKVSDKWYYVNSN | Outside | 0.6212 | Non-Allergen | Non-toxin |
| SDKWYYVNGSGALAV | Outside | 0.552 | Non-Allergen | Non-toxin |
| SDKWYYVNSNGAMAT | Outside | 0.5859 | Non-Allergen | Non-toxin |
| SENTPTVTSSGQDIS | Outside | 1.0498 | Non-Allergen | Non-toxin |
| SQWFKVSDKWYYVNG | Outside | 0.4481 | Non-Allergen | Non-toxin |
| SQWFKVSDKWYYVNS | Outside | 0.4231 | Non-Allergen | Non-toxin |
| SWYYLNANGAMATGW | Outside | 0.6068 | Non-Allergen | Non-toxin |
| SWYYLNANGDMATGW | Outside | 0.6564 | Non-Allergen | Non-toxin |
| SWYYLNSNGAMATGW | Outside | 0.516 | Non-Allergen | Non-toxin |
| TWYYLEASGAMKASQ | Outside | 0.734 | Non-Allergen | Non-toxin |
| WFKVSDKWYYVNSNG | Outside | 0.8744 | Non-Allergen | Non-toxin |
| WYYLNANGAMATGWL | Outside | 0.5875 | Non-Allergen | Non-toxin |
| WYYLNANGSMATGWL | Outside | 0.6401 | Non-Allergen | Non-toxin |
| WYYLNANGSMATGWV | Outside | 0.6793 | Non-Allergen | Non-toxin |
| WYYLNSNGAMATGWL | Outside | 0.4967 | Non-Allergen | Non-toxin |
| WYYVNGSGALAVNTT | Outside | 0.7949 | Non-Allergen | Non-toxin |
| WYYVNSNGAMATGWL | Outside | 0.5124 | Non-Allergen | Non-toxin |

**Table 1.2:** Shortlisted HTL epitopes (CbpA) were further evaluated for IFN-γ production.

| **Epitopes** | **IFN-γ** |
| --- | --- |
| AEGVRSENTPTVTSS | POSITIVE |
| AKVNGSWYYLNANGD | POSITIVE |
| ASQWFKVSDKWYYVN | POSITIVE |
| GGVVHAEGVRSENTP | POSITIVE |
| GSWYYLNANGAMATG | POSITIVE |
| GSWYYLNANGDMATG | POSITIVE |
| GSWYYLNSNGAMATG | POSITIVE |
| KWYYVNSNGAMATGW | POSITIVE |
| LGGVVHAEGVRSENT | POSITIVE |
| NANGDMATGWVKDGD | POSITIVE |
| NANGSMATGWVKDGD | POSITIVE |
| PKTGWKQENGMWYFY | POSITIVE |
| QWFKVSDKWYYVNGS | POSITIVE |
| QWFKVSDKWYYVNSN | POSITIVE |
| SENTPTVTSSGQDIS | POSITIVE |
| SQWFKVSDKWYYVNG | POSITIVE |
| SQWFKVSDKWYYVNS | POSITIVE |
| SWYYLNANGAMATGW | POSITIVE |
| SWYYLNANGDMATGW | POSITIVE |
| SWYYLNSNGAMATGW | POSITIVE |
| TWYYLEASGAMKASQ | POSITIVE |
| WFKVSDKWYYVNSNG | POSITIVE |
| WYYLNANGAMATGWL | POSITIVE |
| WYYLNANGSMATGWL | POSITIVE |
| WYYLNANGSMATGWV | POSITIVE |
| WYYLNSNGAMATGWL | POSITIVE |
| WYYVNSNGAMATGWL | POSITIVE |

**Table 2.1:** Predicted HTL epitopes (PspA) were shortlisted after assessment through TMHMM, VaxiJen v2.0, AllerTOP v.2.0, and ToxinPred servers.

| **Epitopes** | **Transmembrane Helix** | **Antigenicity** | **Allergenicity** | **Toxicity** |
| --- | --- | --- | --- | --- |
| ANGAMATGWAKVNGS | Outside | 0.4051 | NON-ALLERGEN | Non-toxin |
| AAKKDYETAKKKAED | Outside | 1.3549 | NON-ALLERGEN | Non-toxin |
| APAPKPEKPAPAPAP | Outside | 0.6793 | NON-ALLERGEN | Non-toxin |
| APAPKPEQPAPAPKT | Outside | 0.5561 | NON-ALLERGEN | Non-toxin |
| APKPEKPAPAPAPKP | Outside | 0.6494 | NON-ALLERGEN | Non-toxin |
| APKPEQPTPAPKPEK | Outside | 0.6411 | NON-ALLERGEN | Non-toxin |
| DKWYYVNGSGSLAVN | Outside | 0.6870 | NON-ALLERGEN | Non-toxin |
| DKWYYVNSNGAMATG | Outside | 0.6579 | NON-ALLERGEN | Non-toxin |
| GSWYYLNANGAMATG | Outside | 0.6041 | NON-ALLERGEN | Non-toxin |
| GSWYYLNSNGAMATG | Outside | 0.5133 | NON-ALLERGEN | Non-toxin |
| KKDYETAKKKAEDAQ | Outside | 1.4812 | NON-ALLERGEN | Non-toxin |
| KPEKPAPAPAPKPEQ | Outside | 0.6320 | NON-ALLERGEN | Non-toxin |
| KWYYVNGSGSLAVNT | Outside | 0.9912 | NON-ALLERGEN | Non-toxin |
| KWYYVNSNGAMATGW | Outside | 0.7151 | NON-ALLERGEN | Non-toxin |
| PAPAPKPEQPAPAPK | Outside | 0.6432 | NON-ALLERGEN | Non-toxin |
| PAPKPEKPAPAPAPK | Outside | 0.6698 | NON-ALLERGEN | Non-toxin |
| PAPKPEQPAPAPKTG | Outside | 0.7575 | NON-ALLERGEN | Non-toxin |
| PAPKPEQPTPAPKPE | Outside | 0.8065 | NON-ALLERGEN | Non-toxin |
| PKPEKPAPAPAPKPE | Outside | 0.7078 | NON-ALLERGEN | Non-toxin |
| SDKWYYVNSNGAMAT | Outside | 0.5859 | NON-ALLERGEN | Non-toxin |
| SWYYLNANGAMATGW | Outside | 0.6068 | NON-ALLERGEN | Non-toxin |
| SWYYLNSNGAMATGW | Outside | 0.5160 | NON-ALLERGEN | Non-toxin |
| TWYYLEASGAMKASQ | Outside | 0.7340 | NON-ALLERGEN | Non-toxin |
| VNTTVDGYTVNENGE | Outside | 1.5948 | NON-ALLERGEN | Non-toxin |
| WYYVNGSGSLAVNTT | Outside | 0.9395 | NON-ALLERGEN | Non-toxin |
| WYYVNSNGAMATGWL | Outside | 0.5124 | NON-ALLERGEN | Non-toxin |

**Table 2.2:** Shortlisted HTL epitopes (PspA) were further evaluated for IFN-γ production.

| **Epitope** | **IFN-γ** |
| --- | --- |
| ANGAMATGWAKVNGS | POSITIVE |
| AAKKDYETAKKKAED | POSITIVE |
| APAPKPEKPAPAPAP | POSITIVE |
| APAPKPEQPAPAPKT | POSITIVE |
| APKPEKPAPAPAPKP | POSITIVE |
| APKPEQPTPAPKPEK | POSITIVE |
| DKWYYVNGSGSLAVN | POSITIVE |
| DKWYYVNSNGAMATG | POSITIVE |
| GSWYYLNANGAMATG | POSITIVE |
| GSWYYLNSNGAMATG | POSITIVE |
| KKDYETAKKKAEDAQ | POSITIVE |
| KPEKPAPAPAPKPEQ | POSITIVE |
| KWYYVNSNGAMATGW | POSITIVE |
| PAPAPKPEQPAPAPK | POSITIVE |
| PAPKPEKPAPAPAPK | POSITIVE |
| PAPKPEQPAPAPKTG | POSITIVE |
| PAPKPEQPTPAPKPE | POSITIVE |
| PKPEKPAPAPAPKPE | POSITIVE |
| SDKWYYVNSNGAMAT | POSITIVE |
| SWYYLNANGAMATGW | POSITIVE |
| SWYYLNSNGAMATGW | POSITIVE |
| TWYYLEASGAMKASQ | POSITIVE |
| VNTTVDGYTVNENGE | POSITIVE |
| WYYVNSNGAMATGWL | POSITIVE |
