# Supplementary material for: Development of a broad-spectrum epitope-based vaccine against Streptococcus pneumoniae
Source: PLoS One. 2025 Jan 16;20(1):e0317216. doi: 10.1371/journal.pone.0317216 (PMC11737669; doi:10.1371/journal.pone.0317216)
Supplement: S5 Table — (DOCX) [file pone.0317216.s005.docx]

**Table S5:** Disulfide by Design 2.0 predicted probable disulfide bond forming partner.

| Serial no. | Res1 Chain | Res1 Seq # | Res1 AA | Res2 Chain | Res2 Seq # | Res2 AA | Chi3 | Energy | Sum B-Factors |
| --- | --- | --- | --- | --- | --- | --- | --- | --- | --- |
| 1 | A | 22 | ALA | A | 66 | LYS | +73.09 | 2.85 | 0.00 |
| 2 | A | 37 | ALA | A | 44 | GLY | -82.48 | 1.90 | 0.00 |
| 3 | A | 40 | ASP | A | 43 | ALA | +97.29 | 2.90 | 0.00 |
| 4 | A | 51 | VAL | A | 55 | GLY | +89.37 | 5.83 | 0.00 |
| 5 | A | 80 | ALA | A | 179 | ALA | +102.01 | 3.28 | 0.00 |
| 6 | A | 83 | THR | A | 179 | ALA | -108.73 | 4.02 | 0.00 |
| 7 | A | 87 | ALA | A | 92 | TYR | -91.76 | 4.70 | 0.00 |
| 8 | A | 87 | ALA | A | 93 | GLY | -92.00 | 1.13 | 0.00 |
| 9 | A | 88 | ALA | A | 93 | GLY | +69.56 | 3.04 | 0.00 |
| 10 | A | 99 | LYS | A | 168 | TYR | -98.71 | 0.63 | 0.00 |
| 11 | A | 103 | TYR | A | 164 | ALA | -95.21 | 0.36 | 0.00 |
| 12 | A | 106 | ALA | A | 161 | ALA | -95.00 | 1.21 | 0.00 |
| 13 | A | 110 | SER | A | 157 | ALA | -109.52 | 2.94 | 0.00 |
| 14 | A | 113 | ALA | A | 154 | TYR | -89.84 | 0.81 | 0.00 |
| 15 | A | 116 | ASP | A | 150 | ALA | +109.89 | 3.22 | 0.00 |
| 16 | A | 117 | TYR | A | 150 | ALA | -97.91 | 0.97 | 0.00 |
| 17 | A | 120 | ALA | A | 147 | SER | +118.05 | 3.14 | 0.00 |
| 18 | A | 124 | ALA | A | 143 | LYS | -85.93 | 2.36 | 0.00 |
| 19 | A | 127 | ALA | A | 139 | LYS | +120.90 | 5.42 | 0.00 |
| 20 | A | 202 | GLY | A | 305 | ALA | +97.03 | 1.16 | 0.00 |
| 21 | A | 210 | THR | A | 280 | GLY | -100.26 | 7.51 | 0.00 |
| 22 | A | 215 | SER | A | 270 | THR | +101.42 | 1.21 | 0.00 |
| 23 | A | 222 | GLY | A | 264 | GLY | -79.25 | 4.18 | 0.00 |
| 24 | A | 228 | ALA | A | 260 | ALA | +75.79 | 1.74 | 0.00 |
| 25 | A | 231 | ASP | A | 257 | ALA | +104.56 | 4.18 | 0.00 |
| 26 | A | 232 | TYR | A | 257 | ALA | -81.97 | 2.01 | 0.00 |
| 27 | A | 235 | ALA | A | 253 | ALA | +81.82 | 4.48 | 0.00 |
| 28 | A | 239 | ALA | A | 250 | TYR | +86.52 | 3.79 | 0.00 |
| 29 | A | 242 | GLY | A | 246 | GLY | +125.69 | 5.81 | 0.00 |
| 30 | A | 269 | THR | A | 276 | VAL | +82.77 | 5.28 | 0.00 |
| 31 | A | 273 | GLY | A | 293 | GLY | +113.92 | 3.48 | 0.00 |
| 32 | A | 275 | THR | A | 300 | SER | -95.97 | 3.79 | 0.00 |
| 33 | A | 282 | GLY | A | 311 | GLY | +115.59 | 2.81 | 0.00 |
| 34 | A | 285 | GLY | A | 299 | GLY | +69.46 | 4.07 | 0.00 |
| 35 | A | 288 | TYR | A | 296 | GLY | -112.88 | 5.91 | 0.00 |
| 36 | A | 290 | GLU | A | 296 | GLY | -111.52 | 8.24 | 0.00 |
