# Supplementary material for: Development of a broad-spectrum epitope-based vaccine against Streptococcus pneumoniae
Source: PLoS One. 2025 Jan 16;20(1):e0317216. doi: 10.1371/journal.pone.0317216 (PMC11737669; doi:10.1371/journal.pone.0317216)
Supplement: S6 Table — (DOCX) [file pone.0317216.s006.docx]

**Table S6:**

1. Predicted linear epitopes from the vaccine construct.

| **No.** | **Chain** | **Start** | **End** | **Peptide** | **Number of residues** | **Score** |
| --- | --- | --- | --- | --- | --- | --- |
| 1 | A | 26 | 64 | EPAPEPEAEADAEADPEAGIGAVLKVLTTGLPALISWIK | 39 | 0.752 |
| 2 | A | 105 | 160 | AAVKKSEAAKKDYETAKKKAEDAQKKKKYDEDQKKTEAKAKKSEAAKKDYETAKKK | 56 | 0.743 |
| 3 | A | 221 | 267 | SGPGPGAAKKDYETAKKKAEDGPGPGKKDYETAKKKAEDAQGPGPGV | 47 | 0.711 |

1. Predicted discontinuous epitopes from the vaccine construct.

| **No.** | **Residues** | **Number of residues** | **Score** |
| --- | --- | --- | --- |
| 1 | A:M1, A:K2, A:F3, A:L4, A:V5, A:N6, A:V7, A:A8, A:L9, A:V10, A:F11, A:M12, A:V13, A:V14, A:Y15, A:I16, A:S17, A:Y18, A:I19, A:Y20, A:P25, A:E26, A:P27, A:A28, A:P29, A:E30, A:P31, A:E32, A:A33, A:E34, A:A35, A:D36, A:A37, A:E38, A:A39, A:D40, A:P41, A:E42, A:A43, A:G44, A:I45, A:G46, A:A47, A:V48, A:L49, A:K50, A:V51, A:L52, A:T53, A:T54, A:G55, A:L56, A:P57, A:A58, A:L59, A:I60, A:S61, A:W62, A:I63, A:K64 | 60 | 0.764 |
| 2 | A:A105, A:A106, A:K108, A:K109, A:S110, A:E111, A:A112, A:A113, A:K114, A:K115, A:D116, A:Y117, A:E118, A:T119, A:A120, A:K121, A:K122, A:K123, A:A124, A:E125, A:D126, A:A127, A:Q128, A:K129, A:K130, A:K131, A:K132, A:Y133, A:D134, A:E135, A:D136, A:Q137, A:K138, A:K139, A:T140, A:E141, A:A142, A:K143, A:A144, A:K145, A:K146, A:S147, A:E148, A:A149, A:A150, A:K151, A:K152, A:D153, A:Y154, A:E155, A:T156, A:A157, A:K159, A:K160 | 54 | 0.753 |
| 3 | A:I220, A:S221, A:G222, A:P223, A:G224, A:P225, A:G226, A:A227, A:A228, A:K229, A:K230, A:D231, A:Y232, A:E233, A:T234, A:A235, A:K236, A:K237, A:K238, A:A239, A:E240, A:D241, A:G242, A:P243, A:G244, A:P245, A:G246, A:K247, A:K248, A:D249, A:Y250, A:E251, A:T252, A:A253, A:K254, A:K255, A:K256, A:A257, A:E258, A:D259, A:A260, A:Q261, A:G262, A:P263, A:G264, A:P265, A:G266, A:V267, A:N268, A:E278, A:N279 | 51 | 0.689 |
